# Predictors of dental complications post‐dental treatment in patients with sickle cell disease

**DOI:** 10.1002/cre2.335

**Published:** 2020-11-22

**Authors:** Monica Amorim Da Silva Gusmini, Anny Clementino De Sa, Changyong Feng, Szilvia Arany

**Affiliations:** ^1^ Department of General Dentistry, Eastman Institute of Oral Health University of Rochester Rochester New York USA; ^2^ Department of Biostatistics and Computational Biology University of Rochester Rochester New York USA

**Keywords:** complications, number of medications, post‐dentistry, sickle cell disease

## Abstract

**Objective:**

Our aim was to explore potential medical or dental indicators associated with dental complications and the utilization of emergency services in sickle cell disease (SCD), especially that clinical reports on adverse outcomes post‐dental treatment are scarce.

**Materials and methods:**

A retrospective analysis of dental treatments of 47 eligible adults with confirmed SCD between May 2016 and October 2019. Logistic regression analysis was used whether clinical outcomes, course of dental treatment, and regularity of dental care are associated with dental complications after dental procedures and/or resulted in emergency care or hospital admissions.

**Results:**

We identified a new, statistically significant association (*p*‐value = .01) between the number of prescription medications taken and complications (10%) after dental procedures. The most frequent dental procedures were tooth extractions (36%) and pain management (28%) during a non‐scheduled dental encounter (68%). The majority of cases did not participate in regular recall exams and periodical oral hygiene maintenance.

**Conclusions:**

A higher number of prescription medications was associated with an increased risk of post‐dental complications in SCD patients. A thorough medical history, including a list of prescribed medications, and collaboration with the patient medical team are important to assess the risk of complications post‐dental procedures and the need for antibiotic prophylaxis according to the case complexity.

## 1 INTRODUCTION

Sickle cell disease (SCD) is one of the most prevalent genetic disorders worldwide. Studies have reported that approximately 100,000 Americans in the United States are affected by SCD, and one in 365 African American births is affected by sickle cell anemia (SCA) (Brousseau, Panepinto, Nimmer, & Hoffmann, 2010; Ojodu et al., 2014). The prevalence of the disease is highest in sub‐Saharan Africa, Middle East, Southeast Asia, and Mediterranean regions (Piel et al., 2014). SCD is a group of disorders that affects hemoglobin, the molecule in red blood cells that delivers oxygen to cells throughout the body. People with this disorder have atypical hemoglobin molecules called hemoglobin S (HbS), which can distort red blood cells into a sickle or crescent shape (Williams et al., 2009). Normal hemoglobin consists of four proteins subunits: two subunits of alpha‐globin and two subunits of beta‐globin; SCD is developed by the alterations of beta‐globin due to mutations of the gene responsible for beta‐globin production (Kohne, 2011). The presence of two subunits of HbS in human hemoglobin is diagnosed as SCA in affected patients (Ballas & Mohandas, 2004). The sickling may result in irreversible Red Blood Cell modification and decreased oxygen‐transport capacity with circulatory difficulties. The red blood cells' lifespan reduced to 10–20 days instead of 120 days of normal red blood cells, leaving a shortage of red blood cells (Acharya, 2015).

SCD patients can suffer from severe pain crises caused by ischemia or infarction (vascular occlusion) of affected tissues. Dental infections (pulpitis, swelling, dental abscess, and cellulitis) are among the most frequent factors predisposing to vaso‐occlusive crisis (da Fonseca, Oueis, & Casamassimo, 2007; Platt et al., 1994; Ware, de Montalembert, Tshilolo, & Abboud, 2017). Dental professionals should be able to recognize and treat SCD patients with a complete understanding and knowledge of the dental manifestations of this disorder, and consequences of the disease must be considered carefully before dental treatment is started (Kawar, Alrayyes, Yang, & Aljewari, 2018; Laurence, Reid, & Katz, 2002). Previous studies showed that curative treatment is often delayed in patients with SCD (Levenson et al., 2008); thus, aggressive preventive strategies should be implemented to improve oral health and decrease the chances of infection (Javed et al., 2013; Mulimani, Ballas, Abas, & Karanth, 2016). Most of SCD patients are low‐income, socio‐economically vulnerable individuals (Bloom, Simile, Adams, & Cohen, 2012; Luna, Rodrigues, Menezes, Marques, & Santos, 2012) with severe dental issues (Basati, 2014; Demirbas Kaya, Aktener, & Unsal, 2004; Laurence et al., 2002) and without receiving appropriate treatment (i.e., prophylactic cleanings, regular recalls, restorations) when compared to an income‐matched control group of subjects who do not have SCD (Laurence et al., 2006).

Having a dental infection complicated by a sickle cell crisis significantly increases the likelihood of hospital admission among adult SCD patients presenting to the Emergency Department (ED) (Laurence, Haywood Jr., & Lanzkron, 2013; Mulimani et al., 2016). According to relevant literature, patients with SCD are most frequently admitted to hospital emergency care and require subsequent hospitalization compared to other medical conditions. The incidence of oral and dental issues in SCD is higher than that in many other disorders (Acharya, 2015; Alrayyes et al., 2018), but evidence from available clinical studies is very limited for sickle cell patients (Kawar, Alrayyes, Yang, & Aljewari, 2018; Yawn et al., 2014). Previous reports and clinical recommendations highlighted the significance of oral health maintenance by all dental care providers and the development of oral health promotion programs in the communities highly affected with SCD (Laurence et al., 2013; Mulimani et al., 2016; Yawn et al., 2014). However, few data available about the specifics of the current clinical situation in community dental care.

This study aimed to provide evidence‐based information about the dental issues of SCD patients who received dental care through the University of Rochester Medical Centre (URMC) health systems in the Greater Rochester area. Our community‐based dental clinics provide health care in the region of Greater Rochester and Upstate New York, targeting underserved and historically disadvantaged communities, which include patients struggling with SCD. We aimed to examine whether comorbid conditions, medications, and caries prevalence affect the frequency of dental complications and emergency services utilization. This retrospective study aims to identify risk indicators for developing complications post‐dental treatment in patients with SCD.

## 2 METHOD

### 2.1 Data acquisition process

The URMC institutional review board (IRB) approved the research protocol, details of the study aim, data abstraction, chart inclusion, and exclusion (RSRB No. 00003114, approved on December 21, 2018), and the study was performed in accordance with the Declaration of Helsinki. Data were collected about adult sickle cell patients who received dental care through the Eastman Institute for Oral Health (EIOH), URMC, and Strong Hospital affiliated Health Systems in the Greater Rochester area during the period of 05/2016–10/2019. Computerized search using “sickle” and “cell” keywords were programmed by a software analyst at EIOH. SCD specific search and documentation review of existing dental records using the Axium dental software generated an electronic list of potentially eligible patients. Additional information from medical records (eRecords), including patients' engagement with the URMC system in the emergency room, observation unit, and hospital inpatient settings, was obtained. Three reviewers analyzed electronic; medical and dental charts. Prior to data abstraction, the reviewers were trained by careful learning of the variables, the procedural manual, and the data abstraction form (excel sheet). After the initial extraction of a few patient records for practice, discrepancies were reviewed and discussed to clarify any issues. The reviewers analyzed all charts independently based on written agreements of the eligibility criteria, collection method of study variables, and data interpretation. The data used in the statistical analysis were confirmed and approved by the three reviewers.

### 2.2 Inclusion and exclusion criteria

The inclusion criteria were to be diagnosed with SCA (homozygous for HbS) and an adult (19 years old older) who visited the dental clinics of EIOH during the period covered by this review. Both genders and all eligible patients were included in the study. Pediatric and adolescent patients (under the age of 18 years), as well as patients with sickle cell trait (heterozygous for HbS), were excluded from the analysis.

### 2.3 Data retrieval process

A total of 282 dental electronic records were identified in Axium. Cross‐referencing between medical and dental databases (eRecord and Axium, respectively) was done in order to collect the following variables: socio‐demographics (age, gender, race), anxiety (yes/no), pain level, insurance type, medications, and smoking habits. Clinical data extracted from dental records of the EIOH system included the dates of past dental visits and the number of visits at various EIOH locations, the frequency of recall visits, type of treatment, and treatment modifying conditions such as difficulties obtaining satisfactory results of the provided treatment. Data on health service utilization included ED visits, observation unit, hospital admissions, and readmissions within 1 month due to dental issues. Progression and procedure notes created during dental visits were reviewed to reveal details on the dental procedures rendered, including the dental diagnosis (such as infection and swelling), the procedure performed, type of treatment, any noted difficulties obtaining satisfactory hemostasis, additional interventions, and antibiotic premedication. The prevalence of complications of dental origin was reviewed by searching for records of bleeding, infection, and dental pain. Data on health service utilization and complications leading to hospital admission and ED visits were obtained from the hospital records.

### 2.4 Statistical analysis

Due to the rare occurrence of SCD in dental records, the sample size was not calculated as efforts were made to include every eligible subject. Descriptive statistics such as the mean, SD, and median were calculated for the continuous variables. For each categorical variable, the frequency of each category was reported. The association between categorical and ordinal variables was explored using Fisher's exact test. Logistic regression analysis was computed to test the effect of clinical variables on dental complications. An *α* level of <.05 was used to declare significance. Statistical analysis was performed using SAS version 9.4 (SAS Institute Inc, Cary, NC).

## 3 RESULTS

**FIGURE 1:**
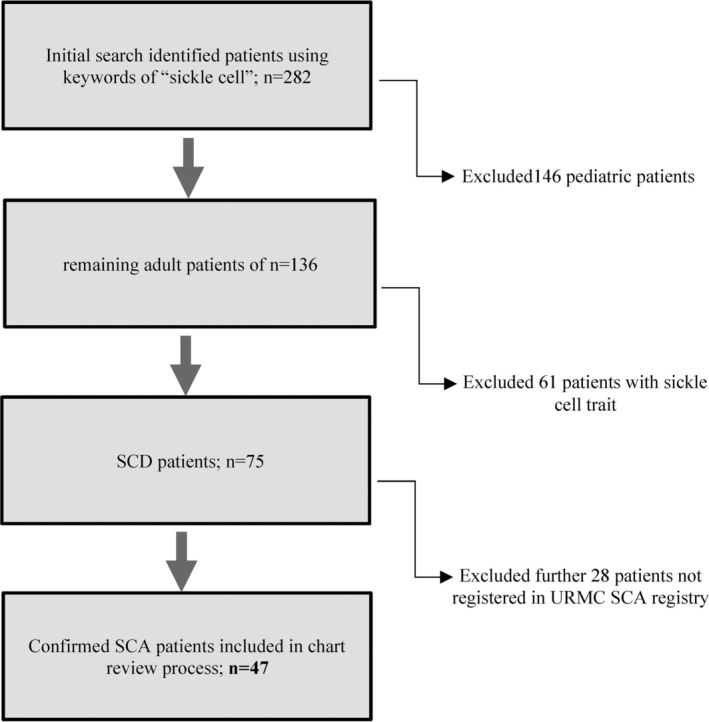
Search strategy and selection process of eligible subjects

Digital dental chart (Axium 7.01.04.56, 1996–2018) software supported searches for the key‐phrase of “sickle cell” identified 282 potentially eligible subjects who visited the EIOH dental clinics between May 2016 and Oct 2019. Once these subjects were extracted from the initial database, the inclusion criteria were applied to further screen patients for study eligibility (see Figure 1). Accordingly, 146 pediatric patients were excluded from the chart review. Patients with sickle cell trait (*n* = 61) and patients with non‐confirmed SCD based on the URMC sickle cell registry data (n = 28) were excluded from the remaining subjects. Finally, 47 subjects with confirmed SCD were included in our retrospective dental and medical record chart review and analysis.

The mean age of patients was 31 ± 9.1 (SD) years, and patients' age was between 19 and 53 years. Thirty‐two (68%) patients were women, and all subjects were African American except for two subjects with Hispanic ethnicity. The descriptive statistics are summarized in Table 1, showing the frequencies of various categorical variables. Most of our patients (78%) visited dental clinics such as urgent care, general dentistry, and other specialty clinics at EIOH. The remaining encounters were reported from the hospital ED and Oral and Maxillofacial Surgery at the URMC with decreasing frequencies in that order. Regular, scheduled visits were recorded for 17 patients, who actively participated in and followed dental recalls for 6‐month hygiene and exam purposes. Forty (85%) patients had federal and/or state‐supported dental insurance (e.g., Medicaid dental plan), four patients had private insurance with dental coverage, and three patients had no insurance at the time of visit.

**TABLE 1:** Demographic characteristics, socio‐economic variables, and the place of encounter for dental care among sickle cell subjects (*n* = 47)

	Number of participants	Percent (%)
Age (years)		
19–29	24	51.1
30–39	14	29.8
40–49	7	14.9
50–59	2	4.3
Gender		
Female	32	68.1
Male	15	31.9
Race		
African American	45	95.7
Other	2	4.3
Ethnicity		
Non‐Hispanic	45	95.7
Hispanic	2	4.3
Smoking status		
Non‐smoker	40	85.1
Smoker	7	14.9
Insurance type		
Medicaid	40	85.1
Private	4	8.5
None	3	6.4
Place of first encounter		
EIOH dental clinic	37	78.7
Emergency department	1	2.1
Oral‐maxillofacial surgery	1	2.1
Dental urgent care	8	17.0
Participants of scheduled dental visits		
Yes	17	36.2
No	30	63.8

Most of the patients (72%) sought dental treatment for dental caries, broken teeth, fractured restorations, which were combined in one category (“defective tooth”) in Table 2. Three patients were diagnosed with a dental infection, and 10 patients had other dental problems, including only one patient with periodontal issues at the time of the first encounter. Extractions were the most commonly performed procedures in SCD patients; every third patient needed tooth removal (17 patients). Extraction was combined with incision and drainage in three cases and with palliative treatment in five patients. The second most frequently rendered treatment was palliative treatment, including a prescription for pain management in 13 patients, followed by filling placements in 12 patients, and cleaning/dental prophy in five patients.

**TABLE 2:** Dental needs and treatments rendered for sickle cell patients (*n* = 47); occurrence of medication usage, opioid prescription, dental pain, anxiety, and complications following dental procedures

	Number of patients	Percent (%)
Chief complaint		
Defective tooth	34	72.3
Infection; dental abscess	3	6.4
Periodontal issues (bleeding, abscess)	1	2.1
Other intraoral problems	9	19.1
Procedure		
Surgical (extraction/with or without other treatments)	17	36.2
Restorative (filling/with or without other treatments)	12	25.5
Palliative (medication prescription and/or referral)	13	27.7
Dental hygiene	5	10.6
Medication usage		
Taking prescription medications (Rx)	47	100.0
Opioid pain management	38	80.9
Opioid prescribed at dental appointment	6	12.8
Antibiotic prophylaxis before dental appointments	7	14.9
Dental pain assessed at the time of appointment		
Yes	13	27.7
No	34	72.3
Dental anxiety assessed at the time of appointment		
Yes	7	14.9
No	40	85.1
Complication after dental procedure		
Yes	5	10.6
No	42	89.4
Type of dental complication		
Bleeding, oozing	1	2.1
Pain	4	8.5
Dental complication required ED visit	2	4.3
Dental complication required hospital admission	3	6.4

All of the patients were taking prescription medications, with an average of 12 drugs (SD ± 6.5), and six patients had over 20 medications prescribed. Despite the fact that 80% of the patients in this study used controlled substances regularly, including opioids for pain management, six (12%) of the patients received opioid analgesics from the dental provider to control pain at the time of dental encounter. Moreover, five of the six patients who received an opioid prescription for relieving dental pain had existing opioid prescriptions for other medical problems.

We searched the medical and dental electronic databases for information on the history of prophylactic antibiotics administration, whether standard protocols for routine dental procedures (Kawar, Alrayyes, & Aljewari, 2018) were applied or whether decisions were made on a case by case basis. We found that prophylactic antibiotic precautions were followed for seven of 47 SCD patients.

Self‐reported dental pain and dental anxiety were documented at the time of dental visit in 27.7% and 14.9% of cases, respectively. Among those who indicated subjective pain level, the visual analog scale (rating of 0–10; increasing order of pain perception) was applied with a median score of 9. For all continuous variables, the mean, SD, and median values are depicted in Table 3, including the caries prevalence for 44 study participants (dental chart was not completed for three subjects).

**TABLE 3:** Descriptive statistics of numerical outcomes including the number of prescription medications taken, dental pain scores, and dental caries status among all subjects

Continuous variable	Number of patients	Mean	SD	Minimum	Maximum	Median
Number of medications taken	47	11.9	6.5	2	29	11.0
Dental pain (VAS score)	13	7.4	3.4	0	10	9.0
Teeth with untreated decay	44	4.3	5.0	0	25	3.0
Missing teeth	44	4.0	4.9	0	22	2.0
Filled teeth	44	6.0	5.3	0	24	6.0
DMFT index	44	15.3	9.4	1	41	13.5

We reviewed Axium and eRecord notes of dental emergencies to determine whether any subsequent complications were associated with dental treatment. We identified five patients (see Table 3) who experienced complications involving persistent bleeding, pain, swelling, and fever with any other unspecified symptoms related to dental treatment within the first month following the dental visit. Complications were oozing in one patient and post‐operative pain in four patients. Among these, two patients had to visit the ED, and three patients were required to be admitted to the hospital due to the complications following dental procedures. No patients developed post‐procedural vaso‐occlusive crisis after dental care.

Regression analysis showed no significant association between regular dental care and the numbers of decayed, missing, and filled teeth (DMFT) (*p*‐value = .59). Smoking (including seven smokers and six former smokers) did not significantly affect the dental caries status, *p* = .31. The effect of anxiety and dental pain on DMFT was investigated, but no significant associations could be detected using Fisher's exact test. When logistic regression analysis was used to study the effect of medications on complications, we found that prescription medications significantly increased the risk of complications after dental treatment (*p*‐value = 0.01) at an odds ratio of 1.3 (1.065 and 1.587; 95% confidence interval). On the contrary, smoking, dental pain, and anxiety did not have a significant effect on dental complications and emergency care or hospital utilization.

## 4 DISCUSSION

The sickle mutation of hemoglobin is the most prevalent inherited, autosomal recessive hematologic disorder, affecting 5% of the world's population and resulting in abnormal changes in the oral cavity and facial structures (Ballas & Mohandas, 2004; Bloom et al., 2012; Booth, Inusa, & Obaro, 2010; Friedlander, Genser, & Swerdloff, 1980; Neves et al., 2012). Several studies found that individuals with SCD have a higher risk of developing dental infection with unknown origins (Mulimani et al., 2016). Accordingly, some research suggested that defective blood flow in the dental pulp of SCD patients can cause asymptomatic changes as well as severe tooth pain with pulp necrosis, without observable clinical pathologies (Basati, 2014; Bishop, Briggs, & Kelleher, 1995; Demirbas Kaya et al., 2004). Since dental problems in SCD often complicate the treatment course of patients (Acharya, 2015; Rada, Bronny, & Hasiakos, 1987), and available clinical data are minimal on dental complications, recent studies emphasized the necessity of clinical investigations targeting communities with high SCD prevalence (Kawar, Alrayyes, Yang, & Aljewari, 2018; Whiteman et al., 2016; Yawn et al., 2014).

The SCD population in Greater Rochester Area is characterized by the following; the majority are African American/Black, and approximately 28% of these patients live in the crescent of poverty. Low socioeconomic status and income disparities are considered factors that may delay patients seeking dental care, and neglecting oral health is common in patients with SCD (Kawar, Alrayyes, Yang, & Aljewari, 2018), who may rely heavily on urgent dental care. A recent study, however, reported that free dental services alone did not improve the occurrence of urgent dental visits (Whiteman et al., 2016). In our study, we observed that most of our patients who sought dental treatment (including all types, such as routine, urgent, or hospital emergency) had federal/state insurance. This was not observed in most earlier studies (Bloom et al., 2012; Luna et al., 2012), which suggested that dental care is often being limited or excluded from health care insurance benefits. As coverage of Medicaid or Medicare assistance in the US has only limited access to primary dental care in many geographical areas, most of the private dental practices refuse to see these patients. Therefore, some of our patients had to travel hours to be seen and access dental care. As transportation issues might limit access to professional care, it would be useful to explore the geographical distribution of SCD patients in this regard.

Regular dental visits are important for controlling odontogenic complications and improve the oral health‐related quality of life of SCD patients (Alrayyes et al., 2018). Therefore, clinical case studies and review papers recommended an “aggressive” preventive strategy in SCD cases, focusing on preemptive procedures including oral hygiene instructions, periodic cleaning, application of fluoride varnish, and avoid more complicated dental treatment needs and higher chances of complications (Javed et al., 2013; Mulimani et al., 2016). Accordingly, improving and maintaining oral health in SCD is the critical component to reducing the risks of severe dental consequences (Kawar, Alrayyes, Yang, & Aljewari, 2018; Laurence et al., 2013). The majority of our patients did not participate in regular recalls or follow‐up treatments, and most of the procedures completed were dental extractions and pain management. One might speculate that patients with SCD have to face exhausting cycles of vaso‐occlusive crisis and might prioritize associated detrimental consequences on their general health (Kawar, Alrayyes, Yang, & Aljewari, 2018; Mann‐Jiles & Morris, 2009). Thus, oral hygiene is often a minor or neglected aspect, as medical issues require more attention. Therefore, implementing dental evaluations at the medical home of SCD patients would be of value to reinforce oral health practices. However, evidence‐based guidelines to treat SCD patients were not published until the 2014 NIH recommendations (DeBaun, 2014; Yawn et al., 2014), which advocated for preventive measures to be coordinated between community healthcare, EDs, hospitals, and other specialists. Despite these recommendations, recent scientific reviews (Acharya, 2015; Kawar, Alrayyes, & Aljewari, 2018) indicated that dental treatment is often delayed in the absence of effective preventive means since the majority of individuals with SCD in the US belong to underserved, minority populations with limited access to dental care. This is a compelling issue, as many dental procedures are not covered by government‐issued insurance.

Assessments of the caries prevalence in SCD patients are rare to find in the literature. The comparison of DMFT indices among those available studies revealed that the mean DMFT index, as well as the numbers of DMFT recorded in our community patients, were very similar to the findings of Kalbassi et al. (Kalbassi, Younesi, & Asgary, 2018), but considerably higher than the numbers reported by Al‐Alawi et al. (Al‐Alawi, Al‐Jawad, Al‐Shayeb, Al‐Ali, & Al‐Khalifa, 2015). The latter study also showed that the number of decayed teeth was more prevalent, and the number of filled teeth was lower among patients with SCD than healthy patients. Both studies investigated younger adult populations (means of 19 and 24 years old, respectively) compared to our study.

Another important point we would like to emphasize is that some patients received opioids for dental pain even though the majority of our patients took controlled substances for pain management regularly. Kawar et al. (Kawar, Alrayyes, Yang, & Aljewari, 2018) suggested that narcotics should be avoided in patients with SCD because they may cause respiratory suppression leading to hypoxia, acidosis, and ultimately a sickle cell crisis. Mulinane et al. (2016) advised prescribing acetaminophen for post‐operative pain control. Furthermore, most studies on SCD‐related dental care suggested the application of prophylactic antibiotics (Smith, McDonald, & Miller, 1987). The study by Kawar et al. stressed that patients with SCD are at higher risk for dental infections than the general population; thus, a prophylactic antibiotic is recommended for invasive dental and major oral surgery procedures. Our study revealed that only 15% of the SCD patients received prophylactic medication, which suggested that case by case decisions dominated patient care, especially in the lack of guidance from Sickle cell disease health organizations.

Poor oral health status and recurrent hospitalizations from dental infections represent special concern in SCD (Kawar, Alrayyes, Yang, & Aljewari, 2018; Mulimani et al., 2016; Williams, Silva, Cline, Freiermuth, & Tanabe, 2018) as dental problems can trigger and complicate the treatment course of vaso‐occlusive crises (Acharya, 2015; Rada et al., 1987). Since available clinical data is very limited to the management of dental complications, we sought to assess the prevalence of dental complications in SCD patients concerning comorbidities and medications. We established a statistically significant, positive correlation between a higher number of medications taken and complications after dental procedures that required visits to the ED or hospital admission. We need to add, though the lower limit of the presented odds ratio confidence interval is close to 1.0, given our small sample size of *n* = 47, the result is significant (*p*‐value = .01). However, we are not aware of any other publications that investigated and reported on the potential dental aspects of the number of medications prescribed in SCD patients. Current data on growing medication use (Gu, Dillon, & Burt, 2010) showed that 20% of the adult population in the US are taking five or more prescription drugs (aka polypharmacy). We speculate that a higher number of medications might be indicating patients with severe underlying medical conditions, which compromised healing and tissue regeneration. As our study showed, 42 of the 47 SCD subjects had polypharmacy, indicating that these patients had several complex medical issues, which contributed to a higher rate of complications after dental procedures were rendered. As a possible explanation, medications are indicators of possible dental complications, rather than the cause. Various medications are known to affect dental treatments and may modify the expected outcomes of dental interventions. Polypharmacy is frequently used in chronic conditions and diseases, and it is documented that the intake of systemic medication may affect regeneration, healing processes (Stavropoulos et al., 2018), and oral health (Barbe, 2018). The chronic application of controlled substances, SCD management drugs, and other medications might be sensitive predictors of complications with treatment; however, further, prospective studies with larger cohort sizes should explore causality relationships.

Statistical evaluation of associations between post‐procedural complications and smoking, anxiety, or dental pain did not yield statistically significant correlations. We were unable to demonstrate a possible correlation between pain and medication use as well. It should be noted that anxiety and pain measurements were rather sporadically recorded among our patients, leading to insufficient data to provide reliable conclusions for statistical analysis. Edwards et al. (Edwards & Edwards, 2010) found that the negative emotional reaction to the somatic experience of pain was predictive of anxiety associated with the onset of painful episodes in diseases like SCD. Besides, patients with SCD are more likely to experience pain than the general population, especially at maxillary sites (Platt et al., 1991; Prevost et al., 2018).We could not find previous studies seeking data on the correlation between smoking and intraoral issues in SCD. While further exploration into the effect of smoking on the oral and periodontal status of SCD patients is needed, Passos et al. (2012) reported recently that SCD does not predispose to periodontal disease. Regarding periodontal problems, our chart review indicated that only one patient received treatment for periodontal issues at the time of encounter. Evidence on this topic is inconclusive, with controversial findings from various reports (Rada et al., 1987) about whether periodontitis can trigger vaso‐occlusive crises and increase the frequency of hospital admissions (Laurence et al., 2013). Two previous clinical studies could not associate SCD with increased levels of gingivitis or periodontitis (Crawford, 1988; de Carvalho, Thomaz, Alves, & Souza, 2016). A future clinical investigation should include assessing of periodontal status, level of oral hygiene, and personal habits (brushing, flossing) of SCD patients.

Our study revealed a 10% rate of complications (mostly pain) after dental procedures. As our retrospective chart analysis and the relevant literature (Brousseau et al., 2010) highlighted, the acute care utilization measured by emergency room visits and hospitalizations was highest for the 18‐30‐year‐old group. This could be explained by the fact that the majority of our patients belonged to this age group, as the demographics of our cohort suggested. Reportedly, SCD patients often rely on repetitive, emergency dental procedures, which contribute to the highest hospital readmissions rates among chronic conditions (Laurence et al., 2013). However, a decrease in hospitalizations for individuals who have received dental care was observed by Whiteman et al. (2016), demonstrating the importance of regular dental care to improve oral health conditions of SCD patients. We investigated other demographic and medical factors likely related to dental attendance and participation in regular dental recalls, but the statistical analysis did not yield significant associations. Research studies addressing the prevention of dental complications from SCD suggested that periodic dental screening (at least every 6 months) provided regularly for patients is the optimal approach to control disease progression (Kawar, Alrayyes, Yang, & Aljewari, 2018). The procedures provided at our community dental offices were mainly guided by curative strategies, indicating the need for site‐specific, preventive treatment protocols for dental aspects of the SCD population. The promotion and elaboration of preventive oral health programs for SCD patients, involving local and community resources, would be ideal for reducing dental complications and the necessity of pain management with opioids.

Limitations of this study include that our data were collected through a retrospective method; thus, the results might reflect the unclear rationale for management decisions and missing variables due to charting omissions. We are also aware that some dental records may have been missed if patients underwent dental treatment at other dental offices before dental care was rendered at our institution. Our relatively small subject number with a convenience sampling technique requires precaution when generalizing the findings. Our chart review revealed that a higher number of medications increased the risk of dental complications in adult SCD patients, highlighting the importance of pharmaco‐medical considerations before the dental treatment of sickle cell anemia. Since our small sample size does not allow us to establish a strong causality relation, we anticipate a stronger correlation in larger scale study. Our findings suggest the possible correlation between dental complications and the number of medications that SCD patients use daily, which may guide future research of this topic on larger cohorts. A multidisciplinary, medico‐dental approach for each patient would help address potential dental complications and improve the oral health‐related quality of life of patients. Attention should be directed at the complex assessment of medical history, with reliable information on each patient's pharmacological management. We also noticed that the majority of cases did not participate in regular recall exams and periodical oral hygiene maintenance. However, all patients with SCD should be encouraged to have their oral health under control by practicing preventive interventions.

## 5 CONCLUSIONS

A higher number of prescription medications was associated with an increased risk of post‐dental complications in SCD patients. A thorough medical history, including a list of prescribed medications, and collaboration with the patient medical team are important to assess the risk of complications post‐dental procedures and the need for antibiotic prophylaxis according to the case complexity.

## CONFLICTS OF INTEREST

The authors declare no potential conflict of interest.

## Data Availability

The data that support the findings of this study are available on request from the corresponding author. The data are not publicly available due to privacy or ethical restrictions.
